# The American Society of Anesthesiologists Physical Status Classification Versus Acute Physiology and Chronic Health Evaluation II for Predicting Perioperative Outcomes in Surgical Patients

**DOI:** 10.7759/cureus.90432

**Published:** 2025-08-18

**Authors:** Laltanpuii Sailo, Saidingpuii Sailo, Rajani Thabah, Jaideep Sonowal, Sunny Aggarwal

**Affiliations:** 1 Anaesthesiology, North Eastern Indira Gandhi Regional Institute of Health and Medical Sciences, Shillong, IND; 2 Anaesthesiology, Zoram Medical College, Aizawl, IND; 3 Anaesthesiology, Divisional Railway Hospital, Dibrugarh, IND

**Keywords:** apache ii score, asa physical status classification, intensive care unit length of stay, perioperative risk stratification, surgical mortality prediction

## Abstract

Background

Accurate perioperative risk stratification guides the management of surgical patients, yet evidence comparing common scoring systems in India is limited. This study aims to evaluate the American Society of Anesthesiologists Physical Status (ASA-PS) classification and the Acute Physiology and Chronic Health Evaluation II (APACHE II) score for predicting perioperative risk, postoperative mortality, and intensive care unit (ICU) use.

Methods

In this prospective follow‑up study conducted at a tertiary care center in Northeast India, adults aged 18-75 years who underwent emergency or elective major surgery between June 2018 and May 2019 were enrolled. Investigators assigned ASA-PS grades and calculated APACHE II scores preoperatively. Receiver operating characteristic (ROC) curves and the area under the ROC curve (AUC) assessed each score’s ability to predict ICU stay > 24 hours and 30‑day all‑cause mortality. Calibration was evaluated with the Hosmer-Lemeshow goodness‑of‑fit test.

Results

Among 152 patients (mean age = 44.7 ± 17 years; 54.6% men), preoperative APACHE II showed better discrimination for prolonged ICU admission (AUC = 0.804) than ASA‑PS (AUC = 0.627). An APACHE II threshold > 7 predicted ICU stay > 24 hours with 75% sensitivity and 74% specificity, whereas an ASA‑PS grade > 2 achieved 69% sensitivity and 50% specificity. For overall mortality, APACHE II and ASA‑PS showed similar performance (AUC = 0.708 vs. 0.737). An APACHE II score > 5 and an ASA‑PS grade > 2 each provided 86% sensitivity, with specificities of 47% and 52%, respectively. Both APACHE II and ASA‑PS showed good calibration (Hosmer-Lemeshow p > 0.05), indicating close agreement between observed and predicted mortality rates.

Conclusions

In surgical patients, the APACHE II score provides a more reliable prediction of perioperative risk and prolonged ICU stay than the ASA-PS classification, while both scores offer comparable, moderate accuracy for mortality. Incorporating objective scoring systems, such as APACHE II, into routine assessment may enhance perioperative decision-making and patient outcomes.

## Introduction

The estimated total number of surgeries performed worldwide is approximately 310 million per year. Between 40 and 50 million surgeries are performed annually in the United States, and approximately 20 million in Europe. Of these, 5% to 15% of patients require readmission to the hospital or the intensive care unit (ICU) within 30 days, and 1% to 4% die. An estimated 8 million deaths occur annually due to major surgeries, making surgery one of the principal causes of death worldwide [1].

The Lancet Commission on Global Surgery set a target of 5,000 surgeries per 1,000,000 population. India currently meets only approximately 30% of this target. While 50 million surgeries are needed annually in India, only 30 million are performed [2]. Timely surgical intervention and accurate prediction of surgical risk and outcomes are essential to saving lives [3].

Meticulous preanesthetic evaluation informs anesthesiologists of a patient’s current medical condition, prior adverse events related to anesthesia or surgery, and expected anesthetic challenges [4]. Standardized preanesthetic evaluation tools, such as the standardized preoperative evaluation form, grading systems, or scoring systems, can improve the quality of information and enhance patient outcomes [5]. A detailed assessment of predicted outcomes is equally important for disease stratification, planning, and treatment, particularly in patients requiring surgery [6].

The American Society of Anesthesiologists Physical Status (ASA-PS) classification system was introduced in 1941 to assess patients’ preoperative condition [7]. Between 1961 and 2020, ASA-PS underwent several revisions to improve its ability to predict perioperative mortality and morbidity [8,9]. Correctly estimating surgical outcomes remains challenging, requiring variables that can be easily calculated both preoperatively and intraoperatively [10].

Evidence-based medicine and advances in statistical indices, such as the Acute Physiology and Chronic Health Evaluation (APACHE), the Physiological and Operative Severity Score for the Enumeration of Mortality and Morbidity (POSSUM), and the Portsmouth-POSSUM, have opened new avenues for predicting risk and outcomes before and after surgery [11,12]. Prolonged ICU stays and poor patient outcomes are associated with increased psychosocial complications and financial burdens for patients and their families. These issues have led to the development of models that quantify costs, resource utilization, patient satisfaction, and case comparisons in mixed populations [13].

This study compares the ASA-PS and APACHE II scoring systems in predicting perioperative outcomes, such as ICU stay, prolonged recovery, morbidity, and mortality. The primary objectives in this study are to compare the predictive accuracy and evaluate the predictive value of both scoring systems. Secondary objectives include identifying risk factors, comparing performance in different patient populations, for example, in elective and emergency surgeries, and evaluating the clinical utility of the scoring systems. The study's results will help clinicians and researchers better understand the strengths and limitations of each scoring system, enabling more effective use in perioperative care.

## Materials and methods

A prospective follow-up study was conducted at a tertiary care teaching hospital in Northeast India. All patients aged 18 to 75 years who required surgery between June 2018 and May 2019 were included in the study. These patients were scheduled for either emergency or routine surgery. Taking into consideration the physiological changes, variability in response to surgical outcomes, and poor socio-economic status of the general population where the study was conducted, patients aged above 75 years were not included in the study. Almost all had comorbidities; some required urgent surgical interventions, while others needed optimization of their physical condition before surgery. Despite substantial surgical risks, all patients underwent surgery after proper evaluation by the operating surgeons. Patients requiring surgical intervention for cardiovascular, neurosurgical, obstetric, or burn-related problems, as well as pregnant or lactating women, were excluded from the study. The major surgeries included in this study were hepatectomy, esophagectomy, pancreaticoduodenectomy, radical hysterectomy, radical cystectomy, radical neck dissection with tumor excision from anywhere in the head and neck area, complex urogynecological surgeries, trauma, and other complicated surgeries were included in the study. The Institution Ethics Committee, North Eastern Indira Gandhi Regional Institute of Health and Medical Sciences approved the study design (Approval No.: P20/17/20).

The ASA-PS and APACHE II scoring systems were used to predict surgical and mortality risk. For planned cases, preoperative anesthetic checkups (PAC) were performed in a designated area by a qualified senior resident and the consultant in charge. Bedside PAC was performed for bedridden patients in the wards and the ICU. For patients requiring emergency surgery, PAC was performed in the preoperative area of the emergency operating theater. Anesthesia consultants referred patients with multiple comorbidities to other departments for further optimization before surgery.

ASA-PS remains the most widely used and convenient grading system for preoperative assessment worldwide. It relies on individual anesthesiologists’ clinical judgment. The system classifies patients into six categories, designated ASA I through ASA VI, with an additional “E” designation for emergency surgeries [7,8]. Specifically, ASA I refers to a normal, healthy patient with no systemic disease. ASA II is assigned to a patient with mild systemic disease. ASA III denotes a patient with severe systemic disease that limits activity but is not incapacitating. ASA IV is for a patient with severe systemic disease that poses a constant threat to life. ASA V is used for a moribund patient who is not expected to survive without the operation. ASA VI is reserved for a declared brain-dead patient maintained for organ donation. The “E” designation can be added to any of these classes (for example, ASA II E) to indicate that the surgery is an emergency.

The APACHE II scoring system predicts surgical risk and the likelihood of ICU stay longer than 24 hours. Mortality risk for surgical patients was calculated within 24 hours of ICU or ward admission. APACHE II scores range from 0 to 71, based on 12 physiological variables, patient age, and underlying health conditions [14]. The 12 physiological variables assessed in APACHE II include temperature, mean arterial pressure (mm Hg), heart rate, respiratory rate, oxygenation (using the alveolar-arterial gradient for inspired oxygen fraction (FiO₂) ≥ 0.5, or the partial pressure of oxygen in arterial blood (PaO₂) in mm Hg when FiO₂ < 0.5), arterial pH, serum sodium and potassium (mmol/L), serum creatinine, hematocrit percentage, white blood cell count, and the Glasgow Coma Scale (GCS) score.

Age-related points in APACHE II are assigned as follows: 0 points for <44 years, 2 points for 45-54 years, 3 points for 55-64 years, 5 points for 65-74 years, and 6 points for >75 years. Chronic health status is scored as 2 points for elective postoperative immunocompromised patients or those with a history of severe organ insufficiency, and 5 points for emergency postoperative patients with severe organ insufficiency [14].

After obtaining informed consent from patients or their legal guardians, detailed medical histories and laboratory investigations were recorded during the perioperative period. Consistent with the approach described by Knaus et al. [14], all patients were scored preoperatively using ASA-PS and APACHE II to evaluate their pre-morbid condition. In this study, both scores were calculated within 24 hours before surgery. Arterial blood gas (ABG) analysis and other relevant investigations were performed preoperatively and postoperatively as advised by the treating surgeon and intensivist.

The ASA-PS and APACHE II scores were used to predict the risk of mortality and morbidity in surgical patients. Patients were followed postoperatively for up to 30 days until discharge or death. Outcomes were measured using parameters such as need for inotropic support, ventilatory support, organ dysfunction, ICU readmission, duration of stay, and recorded morbidity and mortality. Mortality was defined as any death occurring within 30 days postoperatively or as a direct complication of the surgical intervention.

Data were entered into Microsoft Excel spreadsheet software (Microsoft Corporation, Redmond, WA) and analyzed using IBM SPSS Statistics for Windows, version 21.0 (IBM Corp., Armonk, NY). Qualitative variables were expressed as frequencies and proportions, and quantitative variables as means and standard deviations. Independent t-tests were used to assess significant differences between groups. Physiological parameters for APACHE II, expressed as mean ± standard deviation (SD), were assessed preoperatively.

In this study, the APACHE II score was calculated using the 12 physiological variables, age, and chronic health condition.

## Results

A total of 152 patients underwent surgery, including 83 males (54.60%) and 69 females (45.39%). The mean (SD) age was 44.7 ± 17 years, with most patients in the ≤44-year age group, followed by those aged 45 to 54 years. Among these, 98 patients (63%) had elective surgery, while 56 (36%) underwent emergency surgery. Approximately 49 patients (32%) had severe chronic diseases at the time of surgery, including conditions such as cirrhosis of the liver, renal disease, and immunocompromised states (Table 1).

**Table 1:** Baseline characteristics of surgical patients (N = 152). ^a ^Percentages calculated within the subgroup of patients with chronic conditions. Data are presented in the form of frequency (N) and percentage (%).

Characteristic		N	%
Sex	Male	83	54.6
	Female	69	45.4
Age group (years)	≤44	67	44.1
	45 – 54	39	25.6
	55 – 64	29	17.1
	65 – 74	16	10.5
	≥75	3	2.0
Type of surgery	Elective	98	64.5
	Emergency	54	35.5
Chronic health condition	Present	49	32.2
	Absent	103	67.8
Specific chronic condition^a ^(n = 49)	Cirrhosis of the liver	2	4.1
	Renal disease	3	6.1
	Immunocompromised state	44	89.8

Almost all participants were not on ventilator support before surgery, and most (37.5%) required < five days of postoperative ventilatory support. Approximately 5% of participants developed organ dysfunction after surgery, requiring > five days on a ventilator. Longer ventilator duration was associated with increased organ dysfunction (Table 2).

**Table 2:** Ventilator support and acquired organ dysfunction (N = 152). Data are presented in the form of frequency (N) and percentage (%).

Variable	Category	N	%
Preoperative ventilator support	Yes	1	0.7
	No	151	99.3
Postoperative ventilator support	None	80	52.6
		57	37.5
	≥5 days	4	2.6
Preoperative organ dysfunction	Present	1	0.7
	Absent	151	99.3
Postoperative organ dysfunction	Present	8	5.3
	Absent	144	94.7

Approximately 60% of patients stayed in the ICU for ≤ five days postoperatively. The total hospital stay ranged from 11 to 20 days for 48 patients (31.5%), while 29.8% of cases involved stays longer than 30 days. Following surgery, 10% of cases required ICU readmission for postoperative complications (Table 3).

**Table 3:** Postoperative course, hospital stay, and ICU readmission (N = 152). Data are presented in the form of frequency (N) and percentage (%).

Variable	Category	N	%
ICU stay	≤5 days	91	59.9
	>5 days	61	40.1
Total hospital stay	≤10 days	16	10.5
	11 – 20 days	48	31.6
	21 – 30 days	43	28.3
	>30 days	45	29.6
ICU readmission	Yes	15	9.9
	No	137	90.1
Postoperative morbidity	Yes	28	18.4
	No	124	81.6

Most surgical patients were categorized between ASA-PS I and IV. The largest proportions were ASA-PS II (39%) and ASA-PS III (41%), with some patients classified as ASA-PS IV. Only a small percentage were ASA-PS V and VI (Table 4).

**Table 4:** Distribution of ASA-PS grades (N = 152). Ordinal categorical (ASA-PS I to VI) described in frequency (N) and percentages (%). ASA‑PS: American Society of Anesthesiologists Physical Status.

ASA‑PS grade	N	%
I	7	4.6
II	60	39.5
III	62	40.8
IV	16	10.5
V	1	0.7
VI	6	3.9

Physiological variables contributing to the APACHE II score were measured preoperatively, and their means ± SD are shown in Table 5. Oxygenation parameters remained stable, with an alveolar-arterial oxygen gradient of 200 ± 8 mmHg and a PaO₂ of 90.1 ± 9.5 mmHg. Arterial blood‑gas analysis showed a pH of 7.30 ± 0.05. Electrolyte concentrations rose slightly (serum sodium = 142 ± 5.9 mmol/L; potassium = 3.9 ± 0.5 mmol/L), changes expected with the fluid shifts and third‑space losses typical of major or emergency surgery. Serum creatinine also fluctuated, particularly in ill patients (0.9 ± 0.2 mg/dL). Hemoglobin and hematocrit declined modestly (likely reflecting intraoperative fluid replacement or blood loss) while the white blood cell count remained unchanged. The mean APACHE II score was 7.4 ± 2.3, and the GCS fell to 13.2 ± 1.8, indicating a mild deterioration in physiological status during the first 24 hours after surgery.

**Table 5:** Preoperative and postoperative APACHE II physiologic variables. Continuous preoperative variables were summarized as mean ± standard deviation. A–aDO₂: alveolar–arterial oxygen gradient; APACHE II: Acute Physiology and Chronic Health Evaluation II; FiO₂: fraction of inspired oxygen; PaO₂: arterial oxygen tension; SD: standard deviation.

Variable	Preoperative mean ± SD
Heart rate (beats min⁻¹)	89.3 ± 9.7
Temperature (°C)	36.6 ± 0.4
Mean arterial pressure (mm Hg)	87.5 ± 9.3
Respiratory rate (breaths min⁻¹)	19.1 ± 2.1
A–aDO₂ (FiO₂ ≥ 0.50)	200±8
PaO₂	90.1 ± 9.5
Arterial pH	7.30 ± 0.05
Sodium (mmol L⁻¹)	142 ± 5.9
Potassium (mmol L⁻¹)	3.9 ± 0.5
Serum creatinine (mg dL⁻¹)	0.9 ± 0.2
Hemoglobin (g dL⁻¹)	10.0 ± 1.5
Hematocrit (%)	30.1 ± 4.4
White blood cell count (cells µL⁻¹)	9,284 ± 3,780
Glasgow Coma Scale score	13.2 ± 1.8
APACHE II total score	7.4 ± 2.3

**Figure 1:**
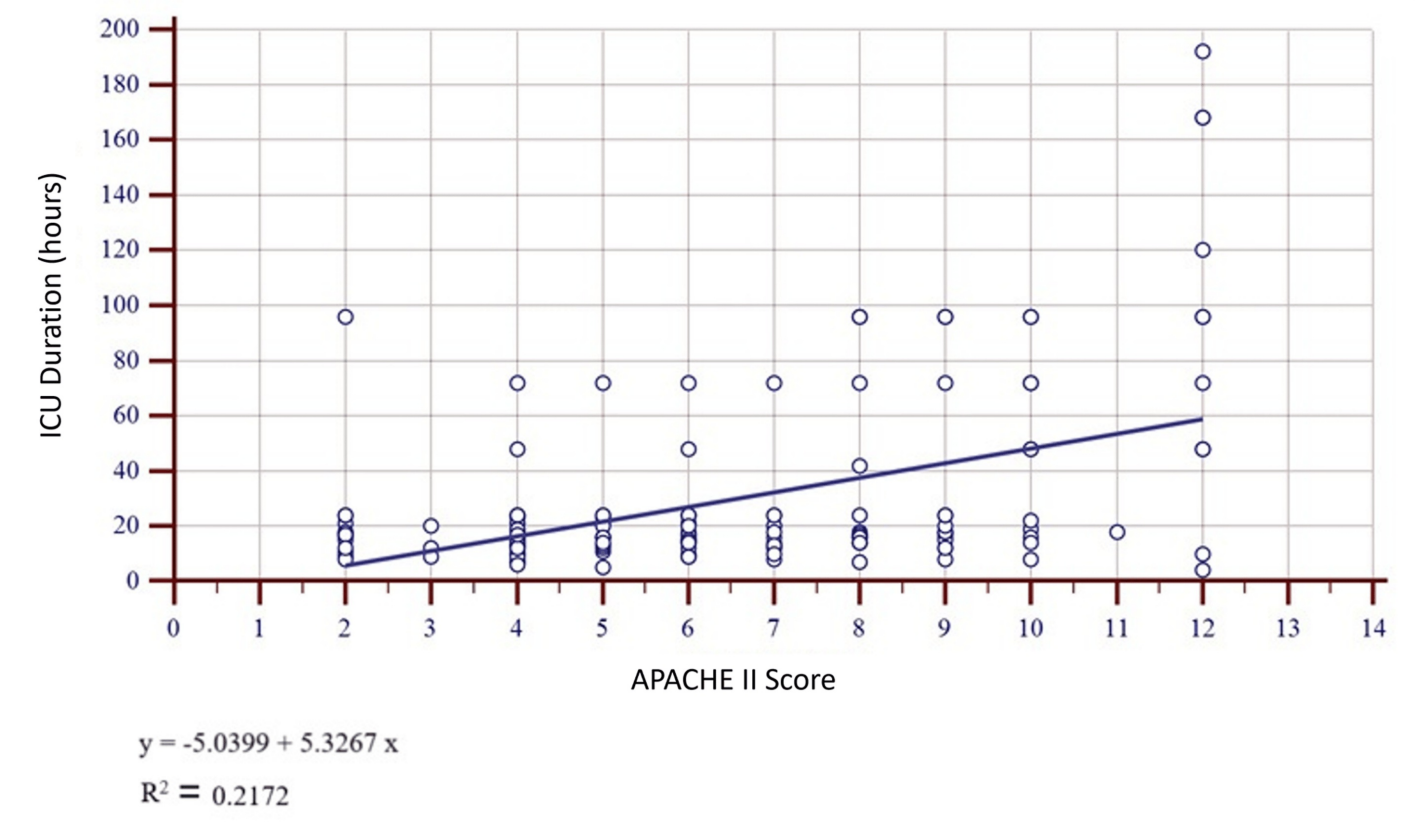
Scatter diagram representing the linear regression relation between preoperative APACHE II scores and duration of ICU stay. APACHE II: Acute Physiology and Chronic Health Evaluation II.

**Figure 2:**
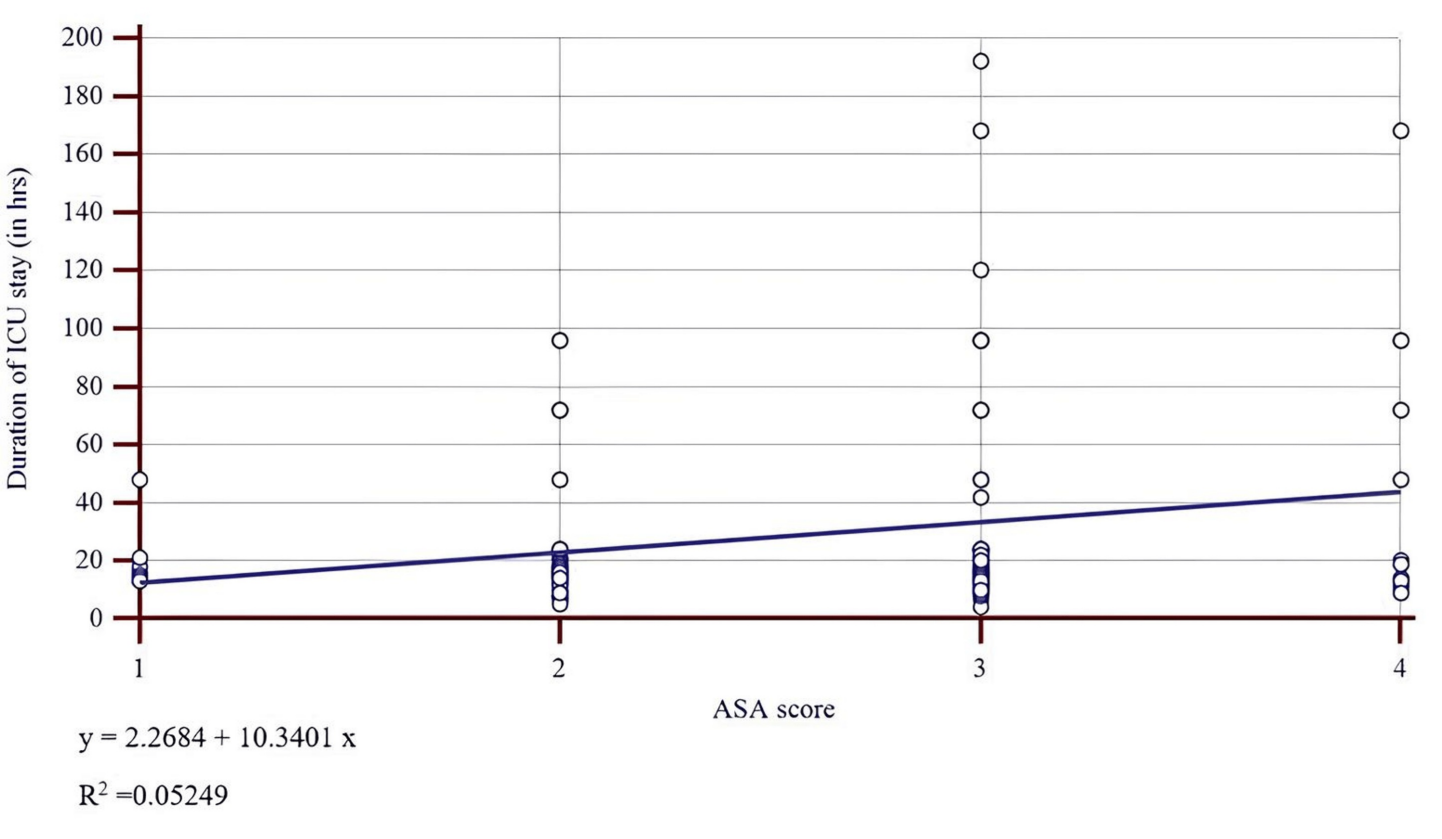
Scatter diagram representing the linear regression relation between preoperative ASA-PS scores and duration of ICU stay. ASA-PS: American Society of Anesthesiologists Physical Status.

Comparison of preoperative ASA-PS and APACHE II scores with ICU length of stay using linear regression (Figures 1, 2 and Table 6) demonstrated a stronger correlation for APACHE II (r² = 0.2) compared to ASA-PS (r² = 0.05). However, both scores showed only weak relationships with ICU length of stay (Table 6).

**Table 6:** Linear regression analysis: preoperative scores vs. ICU length of stay (N = 152). ^a^ Statistical test used: simple linear regression with an F-test for overall model significance. For APACHE II as a predictor of ICU length of stay → p < 0.001 (highly significant). For ASA‑PS as a predictor of ICU length of stay → p = 0.004 (significant). Both are significant, but APACHE-II appears to be more significant than ASA-PS. APACHE II: Acute Physiology and Chronic Health Evaluation II; ASA‑PS: American Society of Anesthesiologists Physical Status; ICU: intensive care unit; SD: standard deviation.

Metric	APACHE II	ASA‑PS
Coefficient of determination (R²)	0.217	0.053
Regression equation (days)	Y = −5.04 + 5.33 X	Y = 2.27 + 10.34 X
Residual SD (days)	28.55	31.41
P-value^a^	<0.001	0.004

**Figure 3:**
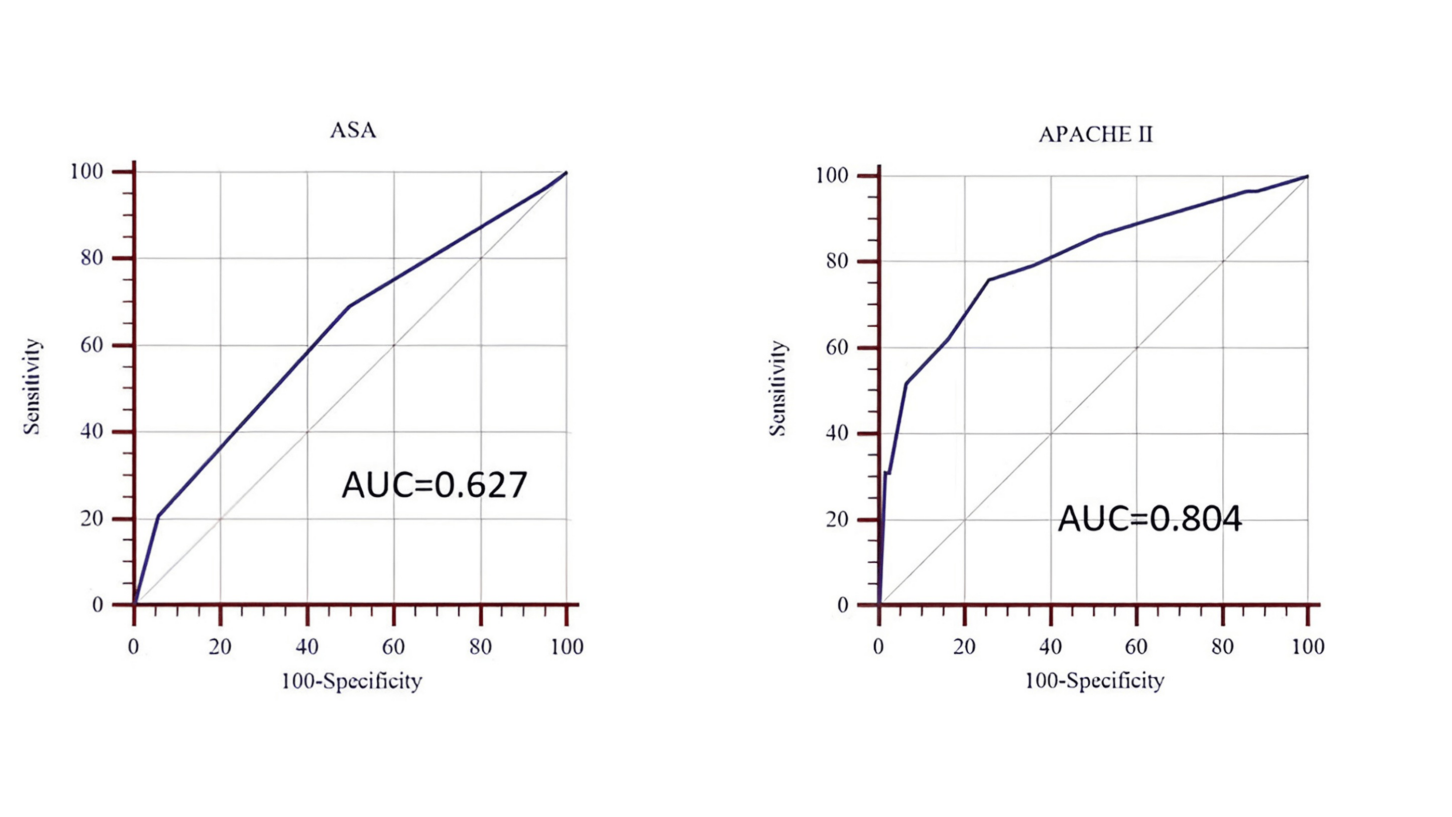
ROC curves of the duration of ICU stay more than 24 hours for preoperative APACHE II and ASA scores. APACHE II: Acute Physiology and Chronic Health Evaluation II; ASA: American Society of Anesthesiologists; ROC: receiver operating characteristic; AUC: area under the ROC curve.

Receiver operating characteristic (ROC) analysis for predicting ICU stay longer than 24 hours revealed that preoperative APACHE II had a better area under the ROC curve (AUC) of 0.804 compared to 0.627 for ASA-PS (Figure 3 and Table 7). An APACHE II score greater than 7 demonstrated 75% sensitivity and 74% specificity for predicting ICU stays exceeding 24 hours, while an ASA-PS score greater than 2 showed 69% sensitivity and 50% specificity (Table 7). APACHE II thus demonstrated superior discriminatory ability with an AUC of 0.804, compared to 0.627 for ASA-PS, indicating that the preoperative APACHE II score is a more reliable predictor of prolonged ICU stay than the ASA-PS score.

**Table 7:** Predictive accuracy of preoperative scores for ICU stay > 24 hours. ^a^ Statistical test: ROC curve analysis. P-values reflect the DeLong test for AUC, and optimal cutoffs were determined with Youden’s index. APACHE II showed good discriminative ability with an AUC of 0.804 (p < 0.001). The optimal cutoff identified by Youden’s index was > 7, yielding a sensitivity of 75.9% (95% CI: 56.5–89.7) and a specificity of 74.4% (95% CI: 65.8–81.8). ASA‑PS demonstrated weaker predictive performance with an AUC of 0.627 (p = 0.004). The optimal cutoff was > 2, with sensitivity of 69.0% (95% CI: 49.2–84.7) and specificity of 50.4% (95% CI: 41.3–59.5). Overall, APACHE II was a significantly better predictor of prolonged ICU stay compared to ASA‑PS. AUC: area under the ROC curve; CI: confidence interval; ROC: receiver operating characteristic; APACHE II: Acute Physiology and Chronic Health Evaluation II; ICU: intensive care unit; ASA‑PS: American Society of Anesthesiologists Physical Status.

Metric	APACHE II	ASA‑PS
Optimal cut-off	>7	>2
Sensitivity % (95 % CI)	75.9 (56.5–89.7)	69.0 (49.2–84.7)
Specificity % (95 % CI)	74.4 (65.8–81.8)	50.4 (41.3–59.5)
Youden index	0.503	0.194
AUC	0.804	0.627
P-value^a^	<0.001	0.004

**Figure 4:**
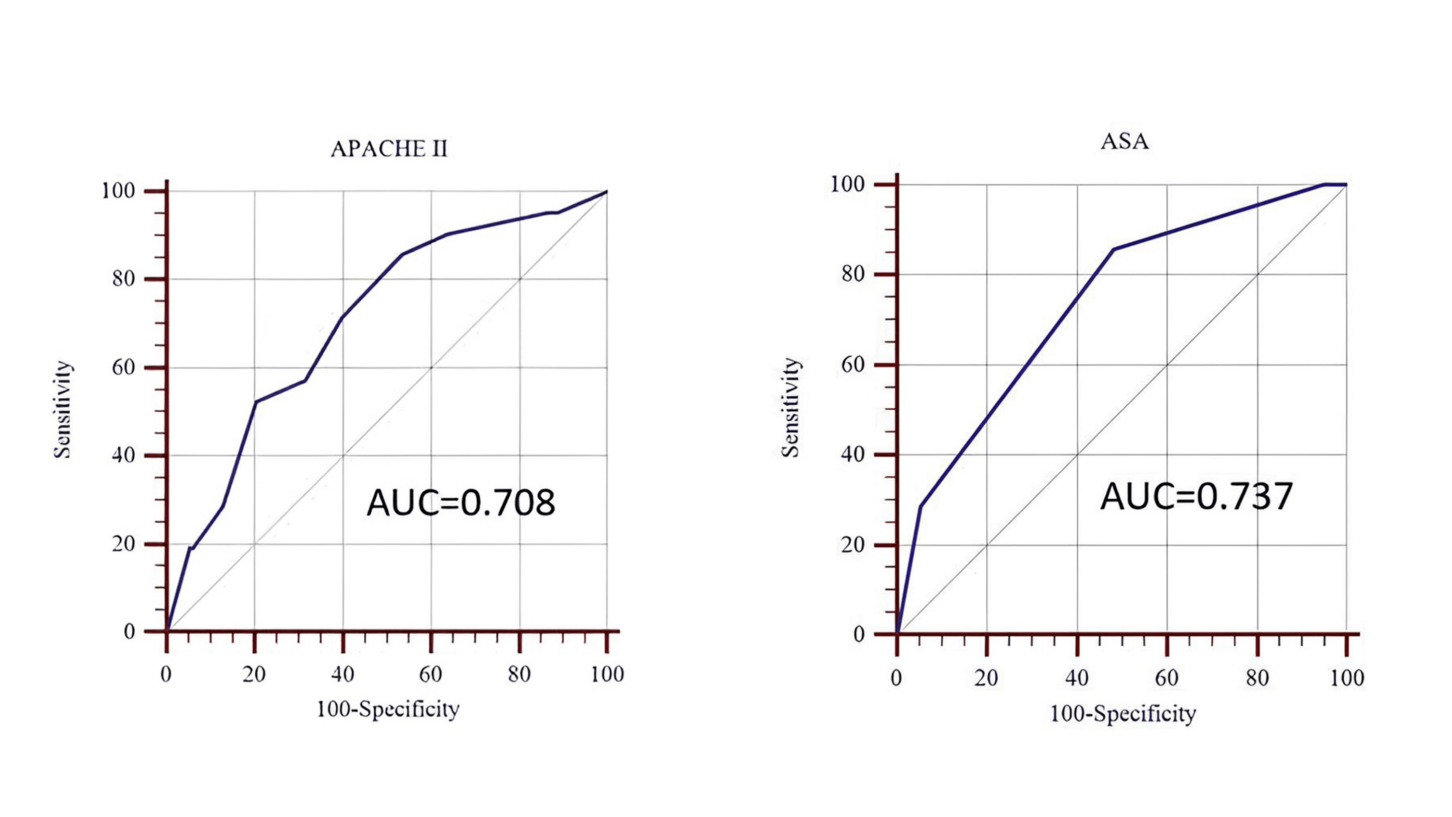
ROC curves of mortality for APACHE II and ASA scores. APACHE II: Acute Physiology and Chronic Health Evaluation II; ASA: American Society of Anesthesiologists; ROC: receiver operating characteristic; AUC: area under the ROC curve.

For overall mortality prediction, preoperative APACHE II and ASA-PS scores had similar AUC values (0.708 vs. 0.737) in ROC analysis. An APACHE II score greater than 5 yielded 86% sensitivity and 47% specificity, while an ASA-PS score greater than 2 had 86% sensitivity and 52% specificity. These findings suggest that both scores provide moderate predictive ability for mortality, though neither offers high specificity, and ASA-PS may perform marginally better in this context. ROC analysis thus showed similar discriminatory performance between preoperative APACHE II and ASA-PS scores for predicting overall mortality (Figure 4 and Table 8). Both APACHE II and ASA‑PS were well calibrated on the Hosmer-Lemeshow goodness‑of‑fit test (p > 0.05), indicating good agreement between observed and predicted mortality (Table 9).

**Table 8:** Predictive accuracy of preoperative scores for 30‑day mortality. ^a^ Statistical test: ROC curve analysis. P-values based on the DeLong test for AUC, with Youden’s index used to identify optimal cutoff scores. P-values: APACHE II: p < 0.001, indicating statistically significant predictive ability. ASA-PS: p = 0.002, also indicating statistically significant predictive ability. AUC: area under the ROC curve; CI: confidence interval; ROC: receiver operating characteristic; APACHE II: Acute Physiology and Chronic Health Evaluation II; ICU: intensive care unit; ASA‑PS: American Society of Anesthesiologists Physical Status.

Metric	APACHE II	ASA‑PS
Optimal cutoff	>5	>2
Sensitivity % (95% CI)	85.7 (63.7–97.0)	85.7 (63.7–97.0)
Specificity % (95% CI)	46.6 (37.9–55.5)	51.9 (43.1–60.6)
Youden index	0.323	0.376
AUC	0.708	0.737
P-value^a^		0.002

**Table 9:** Hosmer–Lemeshow goodness‑of‑fit test for calibration of mortality‑prediction models. * A Hosmer–Lemeshow p‑value > 0.05 indicates good calibration (i.e., no statistically significant difference between observed and expected mortality), so both APACHE II and ASA‑PS accurately reflected the mortality observed in this cohort. APACHE II: Acute Physiology and Chronic Health Evaluation II; ASA‑PS: American Society of Anesthesiologists Physical Status.

Scoring system	Hosmer–Lemeshow χ² (H)	P-value
APACHE II	5.46	0.708*
ASA-PS	8.12	0.420*

## Discussion

Safe anesthetic practice begins with systematic, evidence‑based preoperative evaluation to identify surgical risk and minimize perioperative complications. We applied two widely used tools (the ASA‑PS classification and the APACHE II score) because both can be implemented readily with the resources available in our developing tertiary care center in Northeast India. Menke et al. reported that the routine use of ASA-PS is associated with reduced perioperative morbidity, mortality, and ICU length of stay, supporting its continued use worldwide [15].

APACHE II, originally developed to quantify disease severity and predict mortality in ICU patients, remains a strong predictor of outcome despite the introduction of APACHE III and APACHE IV [11,16,17]. Its relative simplicity permits rapid calculation in emergencies, and several studies have shown that APACHE II outperforms ASA‑PS in outcome prediction [15].

Demographic comparison

The mean age in the present cohort was 44.7 ± 17 years, with most patients aged ≤ 44 years and a male predominance. One‑third had significant chronic disease, and 64% underwent elective procedures. These findings align with reports by Akavipat et al. and de Cássia Braga Ribeiro et al. [18,19]. By contrast, studies by Yurtlu et al. and Fodor et al. described older populations, a higher proportion of women, and a higher proportion of emergency surgeries [20,21], underscoring the variation in baseline characteristics by setting.

Model performance

Robust risk models require discrimination (i.e., the ability to separate survivors from non‑survivors, quantified by the AUC) and calibration, which measure agreement between predicted and observed outcomes [22-24]. Logistic regression in our cohort demonstrated a significant association between the outcome and both pre- and postoperative APACHE II scores, whereas ASA-PS showed weaker correlations. Linear regression confirmed that APACHE II explained more variance in ICU length of stay than ASA‑PS (r² = 0.20 vs. 0.05), although the relationship remained modest.

For prolonged ICU admission (> 24 hours), preoperative APACHE II yielded an AUC of 0.804, compared with 0.627 for ASA‑PS. An APACHE II threshold > 7 provided 75% sensitivity and 74% specificity, while an ASA‑PS grade > 2 offered lower discriminatory power (69% sensitivity, 50% specificity). Thus, APACHE II was a more reliable predictor of extended ICU stay.

Both scores showed comparable performance for overall mortality (AUC of 0.708 for APACHE II vs. 0.737 for ASA‑PS). An APACHE II score > 5 and an ASA‑PS grade > 2 each achieved 86% sensitivity, but specificity remained modest (47% and 52%, respectively). These data suggest that both tools have moderate predictive value for mortality, with ASA-PS showing a slight specificity advantage but APACHE II demonstrating a stronger overall discriminatory ability. Both APACHE II and ASA‑PS were well calibrated on the Hosmer-Lemeshow goodness‑of‑fit test (p > 0.05), indicating good agreement between observed and expected mortality (Table 9).

The mean postoperative APACHE II score in our cohort aligns with the findings of de Cássia Braga Ribeiro et al. [19], who likewise reported physiological deterioration after major surgery. Other authors have reported limited associations between postoperative APACHE II and ICU stay, suggesting an ongoing debate regarding its timing and application [18].

Evolving risk‑assessment strategies

ASA-PS remains popular because it is quick and intuitive; however, its subjectivity introduces inter-observer variability. Emerging biomarkers such as carbohydrate‑deficient transferrin and γ‑glutamyl transferase may refine perioperative risk assessment in patients with heavy alcohol use [24]. Composite indices (including the National Surgical Quality Improvement Program model, the Revised Cardiac Risk Index, and the Charlson Comorbidity Index) may further enhance prognostication [25,26].

Enhanced Recovery After Surgery pathways emphasize prehabilitation and multimodal optimization to reduce pain and functional decline. Recent guidelines from the Indian Society of Anaesthesiologists advocate tailoring preoperative investigations to surgical urgency, comorbidities, and current therapy [27,28]. In the future, user-friendly platforms (potentially incorporating generative artificial intelligence algorithms) could automate complex scores, such as APACHE II, permitting rapid, bedside risk estimation.

Limitations

Our study has several important limitations that should be considered when interpreting its findings. First, it was conducted at a single tertiary care center with a relatively small sample size (152 patients), which may limit its generalizability to other institutions, regions, or healthcare systems. Second, by design, we excluded patients older than 75 years, those requiring cardiovascular, neurosurgical, obstetric, or burn procedures, as well as pregnant or lactating women patients; therefore, the results may not be applicable to these high-risk groups. Third, enrolment was restricted to patients who ultimately proceeded to surgery, introducing potential selection bias because individuals deemed unfit for the operating theater were not analyzed. Fourth, ASA-PS grades were assigned by individual anesthesiologists and are inherently subjective, whereas APACHE II scores were recorded only once within 24 hours of ICU admission, providing limited insight into dynamic postoperative physiology. Fifth, although major comorbidities were captured, unmeasured intraoperative factors, such as anesthetic technique, fluid balance, and blood loss, could confound the observed associations. Finally, outcomes were followed for only 30 days and were not externally validated, precluding assessment of late morbidity or mortality. Larger, multi‑center studies with broader inclusion criteria, serial physiological measurements, and longer follow‑up are warranted to confirm and extend these findings.

## Conclusions

We conducted this study to compare the predictive accuracy of ASA-PS versus APACHE II for perioperative outcomes in surgical patients. In this cohort, the APACHE II score was more accurate than the ASA-PS for predicting preoperative risk, prolonged ICU stay, and postoperative mortality. ASA-PS remains the most widely used classification due to its simplicity, subjectivity, lower predictive power limits, and its utility for detailed risk stratification. Integrating objective, data-driven tools, such as APACHE II, into routine assessment, augmented by modern digital solutions, may improve perioperative decision-making and patient outcomes.

## Appendices

The attached links confirm that the instruments used in the study are freely available:

1. American Society of Anesthesiologists (ASA) Physical Status Classification - official ASA webpage. No license required.

Link 1 - (https://www.asahq.org/standards-and-guidelines/asa-physical-status-classification-system)

This page shows all six classes and carries no licensing restrictions.

Link 2 - ( https://www.ncbi.nlm.nih.gov/books/NBK441940/)

NCBI Bookshelf (StatPearls) has a free chapter titled “American Society of Anesthesiologists Physical Status Classification.”

2. Acute Physiology and Chronic Health Evaluation II (APACHE II)

Link 1 - (https://www.cdisc.org/standards/foundational/qrs/acute-physiology-and-chronic-health-evaluation-ii)

Public‑domain confirmation: the CDISC “Questionnaire, Rating & Scale (QRS) Catalog” lists APACHE II as “Public Domain.”

Link 2 - (PubMed link: https://pubmed.ncbi.nlm.nih.gov/3928249/)

Original description: the 1985 Critical Care Medicine article by Knaus WA et al. (“APACHE II: A Severity of Disease Classification System”) [14].
